# Prognostic value of systemic inflammation response indexes obtained from the complete blood count in patients treated for advanced ovarian carcinoma in front line

**DOI:** 10.1007/s12094-024-03523-3

**Published:** 2024-06-10

**Authors:** Jaime Espinós, José Manuel Aramendía, Antonio González-Martín, Marta Santisteban, Luisa Sánchez, Ángel Vizcay, José Ángel Mínguez, Juan Luis Alcázar

**Affiliations:** 1 Department of Medical Oncology, Cancer Center Clínica Universidad de Navarra, Universidad de Navarra, Madrid, Spain; 2 Department of Medical Oncology, Cancer Center Clínica Universidad de Navarra, Universidad de Navarra, Pamplona, Spain; 3 Department of Obstetrics and Gynecology, Cancer Center Clínica Universidad de Navarra, Universidad de Navarra, Pamplona, Spain

**Keywords:** Ovarian cancer, Systemic inflammation response index (SIRI), Neutrophil-to-lymphocyte ratio (NLR), Platelet-to-lymphocyte ratio (PLR), Monocyte-to-lymphocyte ratio (MLR), Red cell distribution width (RDW), Mean platelet volume (MPV)

## Abstract

**Objective:**

Various systemic inflammation response indexes (SIRI) have repeatedly been described as prognostic factors in ovarian cancer. They have not been validated in prospective trials and published results are sometimes contradictory. We aimed to explore their role in a cohort of patients diagnosed with stage III and IV ovarian cancer treated at our institution.

**Methods:**

We retrospectively examined the prognostic influence of the neutrophil-to-lymphocyte ratio (NLR), the platelet-to-lymphocyte ratio (PLR), the monocyte-to-lymphocyte ratio (MLR), the red cell distribution width (RDW), and the mean platelet volume (MPV).

**Results:**

A total of 77 patients were analyzed. NLR > 2.243 at diagnosis, NLR before primary surgery, MLR at diagnosis, PLR > 289.1 at diagnosis, and PLR at diagnosis were significant in univariate Cox regression for progression-free survival, but none of them retained their significance in the multivariate Cox regression analysis. For overall survival, NLR >  = 2.53 at diagnosis, MLR >  = 0.245 at diagnosis, and PLR >  = 198.3 at diagnosis resulted significant in univariate COX regression; only PLR >  = 198.3 at diagnosis retained its significance in the multivariate analysis.

**Conclusion:**

In our cohort, PLR >  = 198.3 was an independent prognostic factor for worse OS. The definitive role of SIRI in ovarian cancer has not yet been established. If their value as prognostic factors could finally be established, they would become a simple and economical method to predict prognosis in patients with advanced ovarian cancer. Therefore, it is time to conduct prospective, multicenter studies with larger samples to definitively establish its role in ovarian cancer, if any.

## Introduction

According to Globocan, 313.959 women were diagnosed with ovarian cancer worldwide in 2020, with 207.252 deaths registered that year. Most cases are epithelial ovarian cancer (EOC). Despite recent advances, five-year relative survival for the entire group is 38%.

The standard treatment approach for newly diagnosed EOC patients consists of cytoreductive surgery followed by platinum-based chemotherapy, with median overall survival around 41–45 months. Bevacizumab is approved as maintenance therapy but has only demonstrated to prolong the progression-free survival (PFS) with no impact in overall survival (OS) for the intention-to-treat population. Nevertheless, an exploratory analysis of high-risk patients (stage IV or stage III suboptimally debulked) of the ICON-7 trial showed better OS. Recently, PARP inhibitors have changed dramatically the landscape of front-line due to the impact of maintenance treatment on progression-free survival, especially in patients with homologous recombination deficiency regardless of BRCA status (in whom a benefit in OS has been demonstrated also).

It is well-known that chronic inflammation is a risk factor for the development of certain types of cancer [1]. Hanahan and Weinberg included tumor-promoting inflammation as an enabling characteristic of the hallmarks of cancer in 2011. SIRI have been described as simple and inexpensive means to measure patient’s inflammatory state. They have also been described as possible prognostic and predictive markers in ovarian cancer, but results of different studies have not been conclusive [2–7].

The aim of our study is to test the impact of recently described systemic inflammation response indexes (neutrophil-to-lymphocyte (NLR), monocyte-to-lymphocyte (MLR), and platelet-to-lymphocyte (PLR) ratios, mean platelet volume (MPV), and red cell distribution width (RDW) as prognostic and predictive factors in patients treated with advanced ovarian cancer in our institution.

## Patients and methods

### Patients

We retrospectively reviewed the medical records of patients treated with FIGO stage III and IV ovarian, fallopian tube or peritoneal cancer at Clínica Universidad de Navarra (CUN) in the period 2000–2015. Inclusion criteria consisted of patients with histologically confirmed EOC, FIGO stage III or IV, who had surgery (primary or interval cyto-reduction) and first line chemotherapy at CUN. Exclusion criterion was previous diagnosis of infiltrative cancer in the last 10 years except for non-melanoma cutaneous tumors.

## Methods

### Data extraction

All data were extracted from the medical records from the Clinical Records System of CUN.

Primary surgery was performed with the intention to obtain complete cyto-reduction before any systemic therapy. Interval surgery was performed after 3–4 cycles of NAC, and delayed interval surgery after completing 6–8 cycles of primary systemic chemotherapy. Complete cyto-reduction was defined as no macroscopic evidence of residual disease. Surgery complexity was defined using de Aletti’s score. Platinum resistance was defined as disease recurrence < 6 months after the last dose of platinum-based chemotherapy, as this was the definition employed when patients were treated and clinical decisions were adopted according with this definition. The systemic inflammation response indexes explored were as follows: neutrophil-to-lymphocyte ratio (NLR) obtained by dividing the neutrophil count by the lymphocyte count, the platelet-to-lymphocyte ratio (PLR) by dividing the platelet count by the lymphocyte count, the monocyte-to-lymphocyte ratio (MLR) by dividing the monocyte count by the lymphocyte count, the red cell distribution width (RDW), and the mean platelet volume (MPV).

### Statistical considerations

Data were summarized with the number of observations, the mean ± SD, and the 95% confidence intervals for the mean. In ordinal or markedly asymmetric variables, the median with the interquartile range and the 95% confidence intervals for the median were used. The correlation between RDW, MPV, NLR, MLR, PLR and clinicopathological variables was analyzed using the *χ*^2^ test and the Mann–Whitney’s *U* test. ROC (receiver operating characteristic) curves were used for specificity and sensitivity estimates. If the area under the curve was ≥ 0.7, we continued the analysis to find an optimal cut-off. To calculate progression-free (PFS) and overall survival (OS), the Kaplan–Meier method was used. Progression-free survival was defined as the time in months between diagnosis and progression/recurrence according to RECIST 1.1 criteria. Overall survival was defined as the period of time in months between diagnosis and death. Platinum sensitivity was defined as a time interval ≥ 6 month between the last dose, a platinum-based chemotherapy, and disease progression as this was the definition employed when patients were treated and clinical decisions were adopted according with this definition. We used the log-rank and Wilcoxon–Breslow (depending on the Kaplan–Meier survival curve shape) tests to analyze the significance of the differences in survival distribution between groups. In the multivariate analysis, Cox regression analysis was used to determine which biomarkers best predicted disease-free and overall survival. A value of *p* < 0.05 was considered significant. For statistical analysis, STATA software version 15 was used (STATA Corp, Texas, USA).

### Ethical considerations

The study was developed in accordance with the World Medical Association Declaration of Helsinki, with the approval of the Ethics Committee for Research with Medicines of the Community of Navarre, Spain (protocol code: Systemic_Inflamm-Ov-01). Since this study was retrospective, involving no risk for the participants, most patients were death when this analysis was performed and contacting with the families could cause unnecessary suffering, informed consent requirements from the subjects were waived.

## Results

We identified 77 patients who met the inclusion criteria for the period 27/01/2000 to 19/10/2015. Median age at diagnosis was 60 years (IQR 50–67, range 29–82). Median follow-up was 68.8 months (IQR 37.1–120.7, range 4.7–206.3).

Histologies were as follows: 62 (83.78%) high-grade serous, 5 (6.76%) high-grade endometrial, 4 (5.41%) low-grade serous, 2 (2.7%) mucinous, and 1 (1.35%) clear cell. 61 (79.22%) were stage III cases and 16 (20.78%) were stage IV.

The BRCA mutation status was unknown in 57 (72.73%) patients, wild-type in 15 (19.48%) patients, 4 (5.19%) had germline BRCA1 mutations, 1 patient had a somatic BRCA1 mutation, and 1 patient had a germline VOUS in the BRCA1 gene.

A total of 53 (68.8%) patients received primary de-bulking surgery: 47% complete cyto-reductions, 30% cyto-reductions with residual disease ≤ 1 cm. 23 (29.9%) patients received interval de-bulking surgery, 56.5% with complete cyto-reduction, and 34.8% cyto-reduction with residual disease ≤ 1 cm. One patient who received neo-adjuvant chemotherapy had disease progression during treatment and did not received surgery.

After primary de-bulking surgery, patients received a median of 6 cycles of adjuvant chemotherapy (range 6–8), 46.3% with Carboplatin–Docetaxel, 23.9% with intraperitoneal Cisplatin–Paclitaxel, 7.5% with Carboplatin–Paclitaxel, and 22.4% with other protocols.

In the neo-adjuvant chemotherapy group, there were 16.7% complete responses, 70.8% partial responses, 8.3% stabilizations, and 4.2% progressive disease, by RECIST 1.1. Median number of cycles of chemotherapy was 3 (IQR 3–6). 54.2% of these patients received Carboplatin–Docetaxel, 20.8% Carboplatin–Paclitaxel, and 25% other regimens.

The overall response rate (ORR) in 22 patients with evaluable disease by RECIST after the primary de-bulking surgery was 94.1% (72.73% complete responses and 18.18% partial responses).

Median progression-free survival (PFS) for the whole cohort was 21.8 months (CI 95% 18.8–27.7). At 5 years, PFS was 27%.

We performed a receiver operating characteristic curve (ROC) analysis of systemic inflammation response indexes at diagnosis and PFS (Table 1). Patients with NLR ≥ 2.243 at diagnosis had significantly worse PFS, 19.3 (CI 95% 15.97–23.43) vs 43.9 months (CI 95% 20.3–not reached). Patients with MLR ≥ 0.241 at diagnosis had significantly worse PFS, 12.87 (CI 95% 11.63–21.1) vs 17.5 months (CI 95% 12.9–not reached). Patients with PLR ≥ 289.1 at diagnosis had significantly worse PFS, 19.2 months (CI 95%15.5–24.4) vs 24.8 months (CI 95% 18.6–77.5). The AUC for MPV and RDW were 0.52 and 0.42 respectively, so no further analysis was performed.

**Table 1:** Systemic inflammation indexes cutoffs at diagnosis for worse PFS

Index	AUC	Sensitivity	Specificity	PPV	NPV
NLR at diagnosis ≥ 2.243	0.68 (CI 95% 0.56–0.78)	85%	41.18%	83.61%	43.75%
MLR at diagnosis ≥ 0.241	0.72 (CI 95% 0.6–0.8)	88.33%	52.94%	86.89%	56.25%
PLR at diagnosis ≥ 289.1	0.75 (CI 95% 0.63–0.84)	68.63%	3.85%	58.33%	5.88%
MPV at diagnosis	0.52 (CI 95% 0.4–0.64)	–	–	–	–
RDW at diagnosis	0.42 (CI 95% 0.3–0.5)	–	–	–	–

*NLR* neutrophil-to-lymphocyte ratio, *MLR* monocyte-to-lymphocyte ratio, *PLR* platelet-to-lymphocyte ratio, *MPV* mean platelet volume, *RDW* red cell distribution width, *AUC* area under the curve, *PPV* positive predictive value, *NPV* negative predictive value

In the univariate Cox regression model for PFS (Table 2), primary de-bulking surgery (HR 0.35), type of cyto-reduction in primary surgery (HR 0.74), and chemotherapy-induced anemia (HR 0.57) were all protective factors for PFS. NLR at diagnosis > 2.243 (HR 2.41), NLR before primary surgery (HR 1.14), MLR at diagnosis (HR 4.94), PLR at diagnosis > 289.1 (HR 2), and neo-adjuvant chemotherapy (HR 2.82) were risk factors for PFS.

**Table 2:** Univariate Cox regression for PFS

Variable	HR	CI 95%
Neo-adjuvant chemotherapy	2.82	1.75–4.52 (*p* < 0.001)
Primary de-bulking surgery	0.35	0.21–0.57 (*p* < 0.001)
Outcome of cyto-reduction in primary surgery	0.74	0.65–0.85 (*p* < 0.001)
NLR before primary surgery	1.14	1.04–1.26 (*p* = 0.005)
Chemotherapy-induced anemia	0.57	0.34–0.97 (*p* = 0.041)
NLR at diagnosis > 2.243	2.41	1.21–4.81 (*p* = 0.012)
MLR at diagnosis	4.94	1.30–18.76 (*p* = 0.019)
PLR at diagnosis > 289.1	2	1.16–3.46 (*p* = 0.012)

Nevertheless, in multivariate Cox regression, none of these variables retained its significance.

Median overall survival (OS) for the whole cohort was 74.4 months (CI 95% 51.6–123.6). At 5 years, OS was 57% (CI 95% 45.35–67.3).

NLR, MLR, and PLR at diagnosis were significantly associated with OS, so we performed ROC analysis to define cutoffs for worse OS as shown in Table 3.

**Table 3:** Systemic inflammation indexes cutoffs for worse OS

Index	AUC	Sensitivity	Specificity	PPV	NPV
NLR at diagnosis ≥ 2.53	0.67 (CI95% 0.55–0.78)	85.11%	46.43%	72.73%	65%
MLR at diagnosis ≥ 0.245	0.68 (CI95% 0.55–0.78)	91.49%	46.43%	74.14%	76.47%
PLR at diagnosis ≥ 198.31	0.71 (CI95% 0.59–0.82)	76.6%	64.29%	78.26%	62.07%

*AUC* area under the curve, *PPV* positive predictive value, *NPV* negative predictive value

In Table 4, we show the variables that resulted significant in the univariate Cox regression analysis for OS. BRCA mutation status (HR 0.47), primary surgery (HR 0.34), complete primary cyto-reduction (HR 0.3), primary cyto-reduction (HR 0.71), Aletti’s complexity score at primary surgery (HR 0.56), and chemotherapy-induced anemia (HR 0.54) were significantly associated with better OS. NLR at diagnosis >  = 2.53 (HR 3.66), MLR at diagnosis ≥ 0.245 (HR 5.44), PLR at diagnosis ≥ 198.3 (HR 3.38), ASA group at interval surgery (HR 3.81), ascites at primary surgery (HR 5.43), total parenteral nutrition after primary surgery (HR 3.1), and platinum resistance (HR 10.6) were all significant risk factors for death.

**Table 4:** Univariate Cox regression for OS

Variable	HR	CI 95%
BRCA mutation status	0.47	0.25–0.88 (*p* = 0.019)
ASA group at interval surgery	3.81	1.08–13.39 (*p* = 0.037)
Primary surgery yes	0.34	0.19–0.62 (*p* < 0.001)
Primary cyto-reduction	0.71	0.59–0.85 (*p* < 0.001)
Primary complete cyto-reduction vs others	0.30	0.14–0.63 (*p* = 0.001)
Ascites at primary surgery	5.43	1.85–15.86 (*p* = 0.002)
Aletti complexity score at primary surgery	0.56	0.31–0.99 (*p* = 0.047)
Total parenteral nutrition after primary surgery	3.1	1.22–7.90, (*p* = 0.017)
Chemotherapy-induced anemia	0.54	0.30–0.99 (*p* = 0.049)
Relapse < 6 months after platinum	10.6	4.75–23.64 (*p* < 0.001)
NLR at diagnosis > = 2.53	3.66	1.62–8.29 (*p* = 0.002)
MLR at diagnosis > = 0.245	5.44	1.93–15.34 (*p* = 0.001)
PLR at diagnosis > = 198.3	3.38	1.70–6.71 (*p* < 0.001)

PLR was the only systemic inflammation response index with an AUC higher than 0.7 in the ROC analysis for OS. As PLR had high correlation with NLR and MLR at diagnosis (Pearson’s r 0.716 for NLR and MLR, 0.719 for NLR and PLR, and 0.717 for MLR and PLR), we performed a Cox regression model for OS excluding NLR and MLR. PLR at diagnosis >  = 198.3 with HR 3.66, BRCA mutation status with HR 0.35, presence of ascites at primary surgery with HR 4.83, and platinum resistance with HR 7.59 remained as significant factors for OS in the multivariate Cox regression analysis as shown in Table 5.

**Table 5:** Multivariate Cox regression for OS

Variable	HR	CI 95%
BRCA mutation status	0.35	0.12–0.94 (*p* = 0.038)
Primary cyto-reduction	1.3	0.65–2.59 (*p* = 0.45)
Aletti complexity score at primary surgery	0.46	0.19–1.06 (*p* = 0.069)
Total parenteral nutrition after primary surgery	3.62	0.81–16.21 (*p* = 0.092)
Ascites at primary surgery	4.83	1.49–15.63 (*p* = 0.009)
Type of response to adjuvant chemotherapy	1.22	0.59–2.52 (*p* = 0.58)
2nd-look surgery	0.6	0.22–1.68 (*p* = 0.33)
Chemotherapy-induced anemia	0.65	0.23–1.82 (*p* = 0.41)
Relapse < 6 months after platinum	7.59	1.39–41.36 (*p* = 0.019)
PLR at diagnosis > = 198.3	3.66	1.07–12.49 (*p* = 0.038)

Finally, we analyzed the relationship between systemic inflammation response indexes and first relapse < 6 months after last platinum. We found a relation between MLR at diagnosis and first relapse < 6 months with a trend to significance (*p* = 0.055). The ROC curve AUC was 0.7 (CI 95% 0.58–0.8). With a cut-off >  = 0.329, we found a 90% sensitivity, 56.92%, specificity, 24.32% positive predictive value, and 97.37% negative predictive value.

## Discussion

Different SIRI have been described as prognostic and/or predictive factors in ovarian cancer. However, despite the number of reports on SIRI and ovarian cancer, we just found 1 prospective study [8], and one retrospective study with data from a prospective trial [9]. Most studies included patients with different stages (localized and advanced disease) and different histologies (including borderline), several trials did not report multivariate analysis on PFS, OS, or both, and results on the value of SIRI were somehow contradictory. In this context, we considered it pertinent to explore SIRI’s role in stage III–IV epithelial ovarian cancer patients treated at our institution.

In our cohort, PLR was the only SIRI found to be an independent prognostic factor for OS (PLR >  = 198.3 HR 3.66, CI 95% 1.07–12.49). Other 10 studies [10–19] out of 17 that reported a multivariate analysis of OS including PLR found this relationship. Only one of them reported the AUC of the ROC curve, AUC = 0.73 [11], a value similar to our findings.

In our literature review, 10 [3, 10, 12, 20, 21, 11, 22–25] out of 29 studies reporting multivariate OS analysis including NLR did not find it to be an independent prognostic factor, as in our study. Two [24, 26] out of 7 studies did no find MLR to be an independent prognostic factor for OS neither. One study found RDW to be an independent prognostic factor for OS [26].

No SIRI was an independent predictor of PFS in our cohort, not even PLR. In our literature review on NLR, 24 reports included PFS multivariate analysis; as in our cohort, 10 studies did not find a significant association between NLR and PFS [3, 10, 11, 20, 24, 25, 27–30]. Among 13 studies on PLR that reported PFS multivariate analysis, 5 out of 13 did not find a significant association between PLR and PFS [3, 15, 21, 27, 31]. No study to our knowledge reported a multivariate PFS analysis on MPV [4]. Six out of twelve studies on MLR (or LMR, lymphocyte-to-monocyte ratio) reported a multivariate analysis of PFS; 3 out of 6 studies [32–34] found a significant relation between MLR (or LMR) and PFS. None of the two studies that examined the RDW reported multivariate analysis of PFS.

Taking all this information together, we cannot assume that the role of SIRI as prognostic factors in ovarian cancer is a solved issue.

If our results could be prospectively confirmed, we could hypothesize that high PLR levels reflect an imbalance between tumor-promoting inflammation, represented by platelets, and tumor-induced immune response, represented by lymphocytes.

### What does our study add to the current knowledge?

As there are not definitive results in this field, our results add more body of evidence about the possible relation between SIRI and prognosis in ovarian cancer. If these results could be confirmed prospectively, we could dispose of an easy, quick, and cheap prognostic factor that could help in patient’s risk stratification.

### Weakness and strengths

As the majority of studies in this field, ours is a retrospective, single-institution study, with a relatively small sample size. The inclusion period was long, with changes in the pathology reports and in some standard treatments during that period. Despite we examined confounding factors that could influence SIRI like previous infection, use of antibiotics, inflammatory diseases, or use of NSAIs and steroids, we cannot rule out the possibility that different bias can have influenced our results. Due to the small sample size, our study does not have potency enough to generalize these results. All these issues prevent us for making definitive conclusions.

Another weakness is that most of the patients (93% after PDS and 80% in the NAC group) received Docetaxel–Carboplatin scheme instead of the standard Paclitaxel–Carboplatin. For some years, it was elected as the routine regimen in our institution as it had shown similar PFS than Paclitaxel–Carboplatin in the Scottish Gynaecological Cancer Trials Group trial reported by Vasey. The main toxicity of the Docetaxel regimen was neutropenia (which was easily managed with G-CSF) with less grade ≥ 2 neuropathy than the Paclitaxel regimen.

On the other hand, we have a long median follow-up, and we have focused only in stage III and IV disease avoiding the mixture of stages described in other studies that can make it difficult to interpret the results.

## Conclusion

In our study, PLR at diagnosis >  = 198.3 was the only systemic inflammation response index that resulted significant in multivariate analysis for OS, and no SIRI was an independent prognostic factor for PFS. Similar to our studies, most studies reported to date are retrospective, with relatively small sample sizes, mixed populations, and results continue to be conflicting.

The definitive role of SIRI in ovarian cancer is yet to be stablished. If its value as prognostic factors could finally be established, they would become an inexpensive and easy method to predict prognosis, complementing the information obtained from other prognostic factors in patients with advanced ovarian cancer. In addition, its potential role as predictive factor for targeted therapy should be explored. Accordingly, it is time to perform prospective, multicenter, studies with larger sample sizes to definitively establish their role in ovarian cancer, if any. In these studies, the relation of SIRI with other actors in inflammation, like leukocyte subpopulations, cytokines, interleukins, and CTCs, should be explored.

## References

[1] Espinos D, Cigüenza R, Calvo E. 8. INFLAMACIÓN Y CÁNCER. In: Proliferación Celular y Cáncer 2000. Madrid : Fundación Científica de La Asociación Española Contra El Cáncer: Real Academia de Farmacia, D.L. ISBN 84-699-5918-2; 2001:201–221.

[2] Cho H, Hur HW, Kim SW, et al. Pre-treatment neutrophil to lymphocyte ratio is elevated in epithelial ovarian cancer and predicts survival after treatment. Cancer Immunol Immunother. 2009;58(1):15–23. 10.1007/S00262-008-0516-3. 18414853 10.1007/s00262-008-0516-3 PMC11029845

[3] Raungkaewmanee S, Tangjitgamol S, Manusirivithaya S, Srijaipracharoen S, Thavaramara T. Platelet to lymphocyte ratio as a prognostic factor for epithelial ovarian cancer. J Gynecol Oncol. 2012;23(4):265. 10.3802/jgo.2012.23.4.265. 23094130 10.3802/jgo.2012.23.4.265 PMC3469862

[4] Kemal Y, Demirag G, Ekiz K, Yucel I. Mean platelet volume could be a useful biomarker for monitoring epithelial ovarian cancer. J Obstet Gynaecol. 2014;34(6):515–8. 10.3109/01443615.2014.912620. 24832894 10.3109/01443615.2014.912620

[5] Wang X, Yuan Z, Qiu H, et al. The relationship between preoperative blood lymphocyte-to-monocyte ratio and the prog-nostic of epithelial ovarian cancer. Prog Obstet Gynecol. 2016;25(9):654–7.

[6] Li Z, Hong N, Robertson M, Wang C, Jiang G. Preoperative red cell distribution width and neutrophil-to-lymphocyte ratio predict survival in patients with epithelial ovarian cancer. Sci Rep. 2017;7:43001. 10.1038/srep43001. 28223716 10.1038/srep43001 PMC5320446

[7] Baert T, Van CJ, Vanbrabant L, et al. Influence of CA125, platelet count and neutrophil to lymphocyte ratio on the immune system of ovarian cancer patients. Gynecol Oncol. 2018 Jul;150(1):31-37. 10.1016/j.ygyno.2018.05.004. 29751991 10.1016/j.ygyno.2018.05.004

[8] Henriksen JR, Nederby L, Donskov F, et al. Prognostic significance of baseline T cells, B cells and neutrophil-lymphocyte ratio (NLR) in recurrent ovarian cancer treated with chemotherapy. J Ovarian Res. 2020;13(1):1–9. 10.1186/S13048-020-00661-4/TABLES/2. 10.1186/s13048-020-00661-4 PMC7229632 32414391

[9] Marchetti C, D’Indinosante M, Bottoni C, et al. NLR and BRCA mutational status in patients with high grade serous advanced ovarian cancer. Sci Rep. 2021;11(1):1–8. 10.1038/s41598-021-90361-w. 34045513 10.1038/s41598-021-90361-w PMC8159985

[10] Asher V, Lee J, Innamaa A, Bali A. Preoperative platelet lymphocyte ratio as an independent prognostic marker in ovarian cancer. Clin Transl Oncol. 2011;13(7):499–503. 10.1007/S12094-011-0687-9. 21775277 10.1007/s12094-011-0687-9

[11] Zhang WW, Liu KJ, Hu GL, Liang WJ. Preoperative platelet/lymphocyte ratio is a superior prognostic factor compared to other systemic inflammatory response markers in ovarian cancer patients. Tumor Biol. 2015;36(11):8831–7. 10.1007/S13277-015-3533-9/FIGURES/1. 10.1007/s13277-015-3533-9 26063409

[12] Ceran MU, Tasdemir U, Colak E, Güngör T. Can complete blood count inflammatory parameters in epithelial ovarian cancer contribute to prognosis? - a survival analysis. J Ovarian Res. 2019;12(1):1–7. 10.1186/S13048-019-0491-7/TABLES/5. 30744662 10.1186/s13048-019-0491-7 PMC6371536

[13] Miao Y, Li S, Yan Q, Li B, Feng Y. Prognostic significance of preoperative prognostic nutritional index in epithelial ovarian cancer patients treated with platinum-based chemotherapy. Oncol Res Treat. 2016;39(11):712–9. 10.1159/000452263. 27855385 10.1159/000452263

[14] Hu D, Lin Y, Liu F, et al. Elevated preoperative platelet to lymphocyte ratio indicates poor survival in patients with resected high-grade serous ovarian carcinoma. Clin Lab. 2016;62(8):1443–9. 10.7754/CLIN.LAB.2016.151137. 28164624 10.7754/Clin.Lab.2016.151137

[15] Chon S, Lee S, Jeong D, Lim S, Lee K, Shin J. Elevated platelet lymphocyte ratio is a poor prognostic factor in advanced epithelial ovarian cancer. J Gynecol Obstet Human Reprod. 2021 Jun;50(6):101849. 10.1016/j.jogoh.2020.101849. 10.1016/j.jogoh.2020.101849 32619726

[16] Ma XM, Sun X, Yang GW, et al. The platelet-to-lymphocyte ratio as a predictor of patient outcomes in ovarian cancer. A Meta Anal. 2017. 10.1080/13697137.2017.1326894. 10.1080/13697137.2017.1326894 28569074

[17] Zhao Z, Zhao X, Lu J, Xue J, Liu P, Mao H. Prognostic roles of neutrophil to lymphocyte ratio and platelet to lymphocyte ratio in ovarian cancer: a meta-analysis of retrospective studies. Arch Gynecol Obstet. 2018;297(4):849–57. 10.1007/S00404-018-4678-8/FIGURES/5. 29368160 10.1007/s00404-018-4678-8

[18] Tian C, Song W, Tian X, Sun Y. Prognostic significance of platelet-to-lymphocyte ratio in patients with ovarian cancer: a meta-analysis. Eur J Clin Invest. 2018. 10.1111/ECI.12917. 29469190 10.1111/eci.12917

[19] Zhu Y, Zhou S, Liu Y, Zhai L, Sun X. Prognostic value of systemic inflammatory markers in ovarian cancer: a prisma-compliant meta-analysis and systematic review. BMC Cancer. 2018;18(1):1–10. 10.1186/S12885-018-4318-5/TABLES/3. 29669528 10.1186/s12885-018-4318-5 PMC5907305

[20] Thavaramara T, Phaloprakarn C, Tangjitgamol S, Manusirivithaya S. Role of neutrophil to lymphocyte ratio as a prognostic indicator for epithelial ovarian cancer. J Med Assoc Thai. 2011;94(7):871–7. 21774296

[21] Kim HS, Choi HY, Lee M, et al. Systemic inflammatory response markers and ca-125 levels in ovarian clear cell carcinoma: a two center cohort study. Cancer Res Treat. 2015;48(1):250–8. 10.4143/CRT.2014.324. 25761476 10.4143/crt.2014.324 PMC4720074

[22] Feng Z, Wen H, Bi R, et al. Preoperative neutrophil-to-lymphocyte ratio as a predictive and prognostic factor for high-grade serous ovarian cancer. PLoS ONE. 2016;11(5): e0156101. 10.1371/JOURNAL.PONE.0156101. 27203425 10.1371/journal.pone.0156101 PMC4874573

[23] Salman L, Sabah G, Jakobson-Setton A, Raban O, Yeoshoua E, Eitan R. Neutrophil-to-lymphocyte ratio as a prognostic factor in advanced stage ovarian carcinoma treated with neoadjuvant chemotherapy. Int J Gynecol Obstet. 2020;148(1):102–6. 10.1002/IJGO.12986. 10.1002/ijgo.12986 31571212

[24] Chen W, Zhong S, Shan B, et al. Serum D-dimer, albumin and systemic inflammatory response markers in ovarian clear cell carcinoma and their prognostic implications. J Ovarian Res. 2020. 10.1186/S13048-020-00693-W. 32771026 10.1186/s13048-020-00693-w PMC7415177

[25] Liontos M, Andrikopoulou A, Koutsoukos K, et al. Neutrophil-to-lymphocyte ratio and chemotherapy response score as prognostic markers in ovarian cancer patients treated with neoadjuvant chemotherapy. J Ovarian Res. 2021;14:148. 10.1186/s13048-021-00902-0. 34724958 10.1186/s13048-021-00902-0 PMC8561989

[26] Li Z, Hong N, Robertson M, Wang C, Jiang G. Preoperative red cell distribution width and neutrophil-to- lymphocyte ratio predict survival in patients with epithelial ovarian cancer. Nat Publ Group. 2017;7:43001. 10.1038/srep43001. 10.1038/srep43001 PMC5320446 28223716

[27] Zhang H, Lu J, Lu Y, et al. Prognostic significance and predictors of the system inflammation score in ovarian clear cell carcinoma. PLoS ONE. 2017;12(5): e0177520. 10.1371/JOURNAL.PONE.0177520. 28498842 10.1371/journal.pone.0177520 PMC5428928

[28] Farolfi A, Petrone M, Scarpi E, et al. Inflammatory indexes as prognostic and predictive factors in ovarian cancer treated with chemotherapy alone or together with bevacizumab. a multicenter, retrospective analysis by the MITO group (MITO 24). Target Oncol. 2018;13(4):469–79. 10.1007/S11523-018-0574-1/FIGURES/3. 29948780 10.1007/s11523-018-0574-1

[29] Van Nguyen JM, Ferguson SE, Bernardini MQ, et al. Preoperative neutrophil-to-lymphocyte ratio predicts 30 day postoperative morbidity and survival after primary surgery for ovarian cancer. Int J Gynecol Cancer. 2020;30(9):1378–83. 10.1136/IJGC-2020-001378. 32788264 10.1136/ijgc-2020-001378

[30] Farolfi A, Scarpi E, Greco F, et al. Inflammatory indexes as predictive factors for platinum sensitivity and as prognostic factors in recurrent epithelial ovarian cancer patients: a MITO24 retrospective study. Sci Rep. 2020;10(1):1–8. 10.1038/s41598-020-75316-x. 33097745 10.1038/s41598-020-75316-x PMC7585431

[31] Wang YQ, Jin C, Zheng HM, et al. A novel prognostic inflammation score predicts outcomes in patients with ovarian cancer. Clin Chim Acta. 2016;456:163–9. 10.1016/j.cca.2016.03.013. Accessed 1 May 2016. 27006072 10.1016/j.cca.2016.03.013

[32] Gong J, Jiang H, Shu C, et al. Prognostic value of lymphocyte-to-monocyte ratio in ovarian cancer: a meta-analysis. J Ovarian Res. 2019;12(1):1–7. 10.1186/S13048-019-0527-Z/TABLES/3. 31151469 10.1186/s13048-019-0527-z PMC6544921

[33] Lu C, Zhou L, Ouyang J, Yang H, Ding J. Prognostic value of lymphocyte-to-monocyte ratio in ovarian cancer: a meta-analysis. Medicine. 2019. 10.1097/MD.0000000000015876. 31192919 10.1097/MD.0000000000015876 PMC6587575

[34] Cai L, Song Y, Zhao X. Prognostic significance of lymphocyte monocyte ratio in patients with ovarian cancer. Medicine. 2020. 10.1097/MD.0000000000019638. 32243392 10.1097/MD.0000000000019638 PMC7440354

